# Modelling mobile-based technology adoption among people with dementia

**DOI:** 10.1007/s00779-021-01572-x

**Published:** 2021-05-03

**Authors:** Priyanka Chaurasia, Sally McClean, Chris D. Nugent, Ian Cleland, Shuai Zhang, Mark P. Donnelly, Bryan W. Scotney, Chelsea Sanders, Ken Smith, Maria C. Norton, JoAnn Tschanz

**Affiliations:** 1 School of Computing and Intelligent Systems, Ulster University, Londonderry, UK; 2 School of Computing, Ulster University, Londonderry, UK; 3 Department of Psychology, Utah State University, Logan, USA; 4 Department of Family and Consumer Studies, University of Utah, Salt Lake City, USA; 5 Department of Family, Consumer, and Human Development, Utah State University, Logan, USA

**Keywords:** Technology adoption, Medical history, Dementia, Reminder application, Assistive technologies

## Abstract

The work described in this paper builds upon our previous research on adoption modelling and aims to identify the best subset of features that could offer a better understanding of technology adoption. The current work is based on the analysis and fusion of two datasets that provide detailed information on background, psychosocial, and medical history of the subjects. In the process of modelling adoption, feature selection is carried out followed by empirical analysis to identify the best classification models. With a more detailed set of features including psychosocial and medical history information, the developed adoption model, using *k*NN algorithm, achieved a prediction accuracy of 99.41% when tested on 173 participants. The second-best algorithm built, using NN, achieved 94.08% accuracy. Both these results have improved accuracy in comparison to the best accuracy achieved (92.48%) in our previous work, based on psychosocial and self-reported health data for the same cohort. It has been found that psychosocial data is better than medical data for predicting technology adoption. However, for the best results, we should use a combination of psychosocial and medical data where it is preferable that the latter is provided from reliable medical sources, rather than self-reported.

## Introduction

The challenges of caring for an increasing number of the population above 65 are placing a huge burden on existing healthcare services [1]. This burden is further exacerbated by the prevalence of cognitive impairment, which is a common problem observed at this age. Cognitive decline impacts upon efficiency of learning and recall, resulting in slower thinking and poorer memory [1]. These deficits can progressively lead to a more severe types of cognitive disease such as Alzheimer’s disease (AD). In such cases, the affected patient may no longer be able to live independently at home and may require external help to carry out activities of daily living [2]. An urgent rebalance between the needs of older patients with cognitive impairment and the available health facilities is essential to combat this burden and reduce costs. Assistive technologies are one potential solution for the provision of elder care. These technologies, in general, have the capacity to improve quality of life and enhance independence of its users. However, the acceptance of assistive technologies is critical to their success. Abandonment of assistive technologies is a common concern among technology developers and healthcare professionals. To help make positive changes in the life of people with dementia (PwD) through the aid of technology, it is important to understand the reasons that can impact on the widespread acceptance of such technologies. Understanding the reasons that contribute to a lack of adoption of assistive technology is crucial to their success in the long term. The aim of this research is to carry out an early-stage evaluation of assistive technologies and analyse the reasons that affect their adoption in a cohort of older adults with cognitive impairment.

With the aim of addressing the increasing demands of elder care, independent living research has emerged to support older adults through technology-based assistance [2]. Assistive technologies bring intelligence to the surroundings and proactively support users with daily activities. Examples of such technologies comprise of assistive solutions that can help to carry out activities of daily living, facilitate remote monitoring, prompt tasks, and encourage social interactions [3]. These assistive technologies are, however, useful only if they are adopted. A key requirement is to understand the typical or expected lifestyle of the individual and accordingly suggest relevant solutions based on the subject’s current cognitive abilities and willingness to adopt the technology. Therefore, a basic prerequisite in designing an assistive technology solution is to have a basic understanding of the reasons that are critical to the user’s decision in adopting these solutions.

Previous research on assistive living has overlooked the reasons for the abandonment of assistive technologies in the long term. Predicting the likelihood of adoption could be beneficial in an early stage evaluation of assistive technologies success. Such evaluation can be helpful in avoiding negative impacts and associated distress due to failure among PwDs [4–8]. Further, if a particular technology is found to be unsuitable, an alternative solution of caregiving and the level of caregiving provided to a PwD can be provided accordingly. In addition, such early prediction will help in reducing the associated cost of deployment of these assistive technologies inappropriately. Incorporating of such information in learning adoption, decisions have proved to be beneficial and are a relatively new area of research. Recent research has been directed towards studying reasons that relate to the adoption and abandonment of technology [9, 10].

To minimise the likelihood of failure of assistive technologies, our research aims to understand the potential user’s background before recommending assistive technology and predicting long-term adoption success. The modelling of adoption is to be done by analysing the potential users’ background, psychosocial, medical history data, and technology usage, thus personalising the provision of assistive care. The Technology Adoption and Usage Tool (TAUT) project [11] is designed with keeping above view in mind. The TAUT project aims to develop an adoption prediction tool that can give an insight into a PwD’s willingness for adoption. The objectives of the TAUT project are described below:

1. Find features that could provide insight into the PwD’s likelihood to adopt an assistive technology
2. Identify features that support or do not support adoption prediction and forecast adoption success
3. Approximate the underlying function and find a correlation between the output class and the input features, while keeping the size of the resulting model small and easy to interpret
4. Classify adopters from non-adopters accurately based on the identified set of features.

**Fig. 1:**
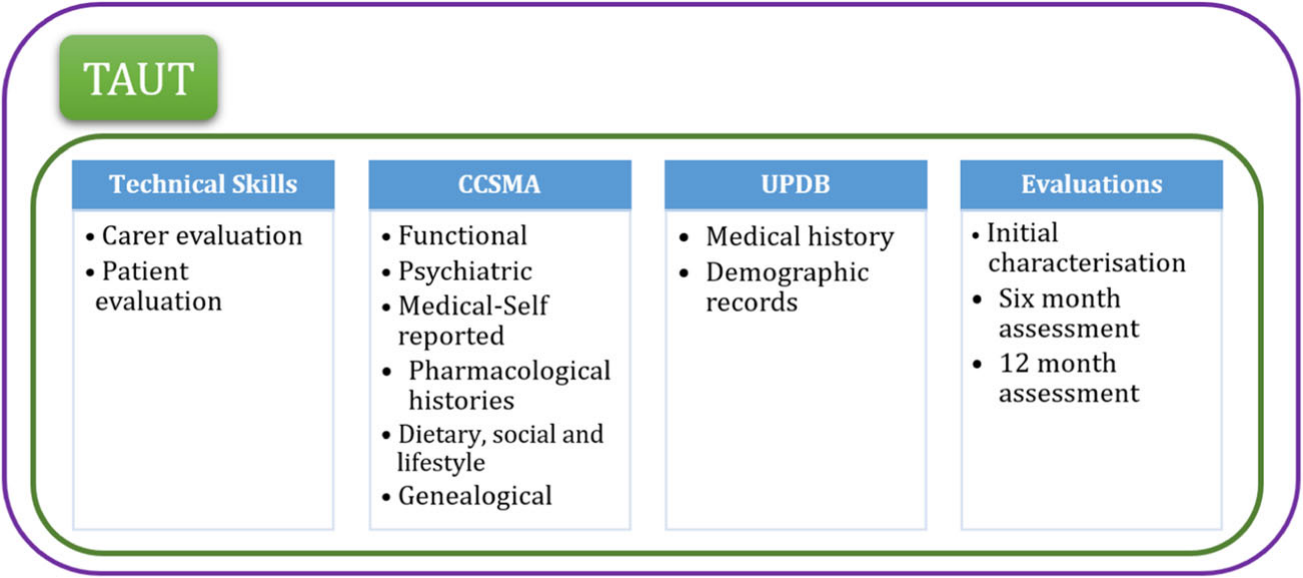
An overview of the TAUT, indicating sources of the data and set of features used in the project

The focus of our work is directed towards increasing the understanding of adoption success and including features that are more informative. The prediction tool is to be implemented within TAUT to classify adopters and non-adopters based on usage of assistive technology in the form of a reminder application (app). A set of features including psychosocial and medical history data is used as input into the prediction model. Through collaborations with the Utah State University and University of Utah, data from Cache County Study on Memory in Aging (CCSMA) [12] and Utah Population Database (UPDB) [13] is obtained. The TAUT project recruited participants from CCSMA to provide a 12-month assessment of a TAUT reminder app [14]. The subjects in the CCSMA dataset are linked to the UPDB dataset, which provided medical history data of the subjects. It is envisioned to build the final adoption model using data related to participants’ compliance with reminder app usage and details of psychosocial and medical history data. The CCSMA dataset contains personal information, details of cognitive assessments, and self-reported medical data that are collected through sessions with the PwDs and their caregivers, while the UPDB dataset contains medical history data obtained from healthcare insurers. Figure 1 details different sources of data in the TAUT study. These are explained in greater detail within the methods section of this paper.

Since the TAUT project is dealing with three different types of datasets, the model building and feature selection process has been carried out with each of these three datasets separately. The first stage analysed only the CCSMA data and reported 11 features that are more likely to be associated with the adoption, achieving an accuracy of 92.48% [4]. In the second stage, initial analysis is carried out using only the medical history features from the UPDB. The UPDB analysis achieved an adoption prediction accuracy of 85.80% with a set of 24 features [5]. Further details of both these results are described later in this paper. It is to be noted that the medical history information in the UPDB comes from health sources, whereas the CCSMA dataset contains self-reported medical history along with other psychosocial features. While the data from the UPDB dataset is more readily accessible than that from the CCSMA dataset, the results from CCSMA highlight the advantage of incorporating personal and background information within the adoption prediction model. The work described in this paper is the third stage of this work. In the current work, initially, the feature selection is carried out on the 24 features from the UPDB dataset. Following this, the CCSMA and UPDB features are merged. Feature selection is carried out on the merged dataset to find the subset of features that are more likely to be associated with technology adoption. Finally, the adoption model is built using the selected CCSMA and UPDB dataset features.

## Related work

Assistive technology acceptance is crucial for its success in elder care [15]. With the availability of a vast range of technology and diversity in users’ background, it is important to understand the reasons that affect adoption [16]. To understand the reasons that influence adoption, the technology acceptance model (TAM) [17] and the psychosocial impact of assistive device scale (PIADS) [16], have been developed within the literature. TAM is an information system theory, which models how subjects use and accept new technology. TAM has been used as a tool for assessing the success of new technology. TAM is built on reasoned action and states that subject’s behaviour is affected by usefulness and ease of use. TAM defines six domains: demographic variables, perceived usefulness, perceived ease of use, attitudes towards use, behavioural intention, and actual use as determining factors [18]. In general, personal information and social aspects impact the acceptance of the technology. However, TAM does not incorporate factors for social influences, and this stands as a major limitation [19]. PIADS is an extension of TAM and includes personal factors as well as external factors such as people and society that may affect usage and self-image. PIADS has been used for assessing assistive technology adoption [16]. However, both TAM and PIADS have been criticised for lack of consideration of explanatory behaviour and experimental evaluation [19]. As an improvement on both approaches, the unified theory of acceptance and use of technology (UTAUT) [20] has been developed. UTAUT identifies more reliable features in the model and includes features such as user expectations of the technology and willingness to use it, as well as age and gender. For understanding mobile phone usage and adoption by older patients, a TAM-based Senior Technology Acceptance & Adoption model for Mobile technology (STAM) was developed in [6].

Recent studies have found that people in different age groups think differently when it comes to making decisions about technology adoption and its usage [7]. There is an increasing interest in understanding causes that affect older patients’ decision of adoption [21, 22]. Generally, older patients consider that technology brings progress and convenience; however, they are doubtful about its benefits considering their skill sets [23], anxiety in using such technologies, and lower self-efficacy [24]. It has also been found that older patients are more interested in technologies that are simple, convenient, and easy to use with added safety and security [7]. Mobile phones and other related technologies may be status symbols for some, but older users might still not adopt. A positive impact on older patients is often associated with how technology sustains their daily activities [23]. There can be conflicting views regarding perceived usefulness between technology developers and older patients.

Limited research has been carried out in understanding the reasons that affect technology adoption in cognitively impaired older adults. Previous work has specifically been focused on understanding general reasons related to adoption; however, predicting long-term adoption has been widely ignored. In [18], a framework for modelling the selection of assistive technologies is developed to identify the best fit assistive technology for PwDs; however, the framework is only used to select an assistive technology without tackling the need to consider long-term adoption. Reasons related to the rejection of assistive technologies are identified in [23], which included easy procurement, lack of user opinion in design and development, poor performance, and changes in user needs. These outcomes indicate that with improved service design, user satisfaction can be increased while reducing rejection rates. Thus, from the adoption point of view, it is essential to include features such as user’s background information such as employment, education, and medical history. Hence, the TAUT project explores different datasets containing these features to analyse reasons for rejection or adoption. Another innovative aspect of the TAUT project is not to rely on complex questionnaires about perceived utility and use but include generally easily obtainable demographic and health information. Table 1 compares different technology acceptance models built and the proposed TAUT project.

**Table 1:** Summary table comparing different technology acceptance models built and proposed

Models and theories	Description
TAM [17]	Based on perceived usefulness and ease of use are key to the acceptance of the technology. Lacks explanatory and predictive power. No experimental evaluation is done and does not include social influences
PIADS [16]	Extension of TAM and incorporates personal factors and external factors like people and society. Criticised for lack of consideration of explanatory behaviour and experimental evaluation
UTAUT [20]	Improvement on TAM and PIADS. Identifies more reliable features like age, gender, willingness to use, user expectations, and willingness to use. Also, includes facilitating conditions (infrastructure) as a determining factor
STAM [6]	Developed to understand mobile phone usage and adoption by older patients. More related to mobile phone usage in general and not evaluated for assessing assistive technology adoption
Scherer et al. (2007) [18]	Framework for modelling the selection of assistive technologies. Used only for selecting an assistive technology and does not consider long-term adoption
TAUT [4]	The proposed TAUT model investigates factors that affect technology adoption in the cognitively impaired older cohort. Range of features used such as subject’s background, environmental and social perspectives, and medical history information are used. The proposed adoption model is to be built by using information related to users’ compliance with the TAUT app usage, users’ background, details of cognitive assessment, and medical history data. Does not rely on complex questionnaires but include generally easily obtainable demographic and health information

## Methodology

To understand the reasons for successful adoption or rejection, our objective is to glean a more detailed set of information that can give insight into a PwD’s potential for adoption and possible reasons for rejection. Hence, in the TAUT project, we have three sets of information: (1) information relating to individuals’ compliance with usage of the TAUT reminder app, (2) details of user’s personal and background information from the CCSMA, and (3) medical history data from the UPDB. Before detailing the data collection, a brief overview of TAUT reminder app is detailed below.

### TAUT reminder app

**Fig. 2:**
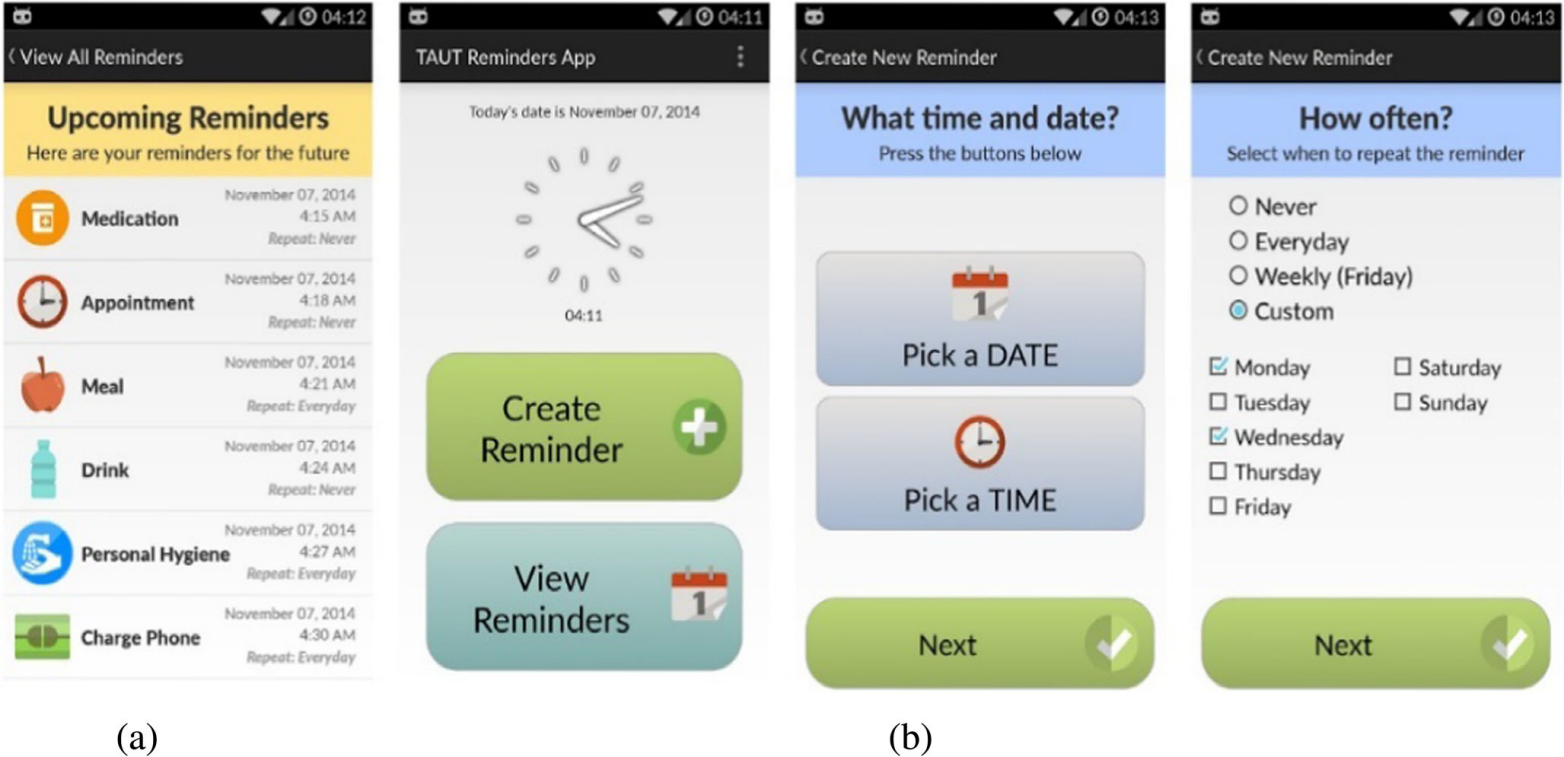
Screenshots from the TAUT app showing (**a**) upcoming reminders list and (**b**) reminder creation screens

The TAUT reminder app benefits from 10 years of knowledge in the design, execution, and assessment of assistive reminding solutions [14]. The app has been designed by an interdisciplinary team following an iterative design process and has been earlier evaluated, with a representative cohort, on a small scale [25]. The current form of the app, described in [14], is an android-based app and offers a user interface to schedule and acknowledge reminders. Reminders can be scheduled for a range of activities of daily living such as meals, appointments, medication, and bathing. Figure 2 shows screenshots from the TAUT reminder app. The reminders can be set by a PwD, by a caregiver, or a family member. The reminders are delivered at the time specified and presented as a popup dialogue box on the screen accompanied by a picture indicating the type of activity, a textual description, and a melodic tone. If the reminder is acknowledged within 60 s, the reminder is logged as “acknowledged”; otherwise it is logged as “missed”. The app has the additional functionality of recording the audio messages.

The TAUT reminder app records details of user’s interactions such as what time reminders are scheduled, when they are acknowledged, the number of times missed, and type of reminder. These details will be used later to analyse how well the user is engaging and adopting the app. The use of the application will give details of which activities the user requires more assistance, the form in which they prefer reminders (voice or text), and the common times for reminders [14].

### Data collection

The CCSMA is a population-based, longitudinal study of AD and other dementias, which has followed over 5000 elderly residents of Cache County, Utah (USA) since 1995 [12]. The residents included approximately 1033 individuals aged 60 years and older. In the CCSMA study, every 3 years, periodic waves of visits are performed to update the information. The study was created to reflect on genetic and environmental factors related to risk for AD and other forms of dementia [4]. Additionally, the CCSMA database is linked to the UPDB dataset at the University of Utah, which includes participant’s medical, genealogical, vital signs, and demographic records [11]. The UPDB dataset is updated annually with complete reporting of medical information for the past 20 years. The participants in the CCSMA represented the cognitively impaired older cohort appropriately and could be a potential user of TAUT reminder app. Therefore, the TAUT project actively recruited participants from the CCSMA to provide a 12-month assessment of a TAUT reminder app [14].

Individuals are categorised as non-adopters at different levels as (1) individuals interested in trying the TAUT app, but incapable of using it for some reasons, (2) individuals who are capable but disinterested in trying the TAUT app, and (3) individuals who are incapable and disinterested. Table 2 presents the user adoption matrix showing adopters and non-adopters. The capability of individuals is assessed through questionnaires during the enrolment process.

**Table 2:** User adoption matrix profiling adopters and non-adopters based on capability and willingness

Adoption modelling		Capability	
		Yes (proficient)	No (non-proficient)
Willingness	Yes (interested)	Adopter	Non-adopter (1)
	No (not interested)	Non-adopter (2)	Non-adopter (3)

The work described in [11] details the TAUT participant recruitment process. The number of successful adopters has changed through the life course of the study as participants dropped out or passed away. The work reports that initially 335 subjects are screened and contacted by mail. At this stage of the recruitment process, 51 refused to participate (non-adopter 2) and 55 are deceased. The research team assessed the remaining 229 subjects via telephone. Out of these 229 subjects, 98 are unreachable, 90 refused (non-adopter 2), and 41 agreed to participate. Out of these 41 participants, 12 are currently enrolled, 9 agreed to participate but require screening, 18 successfully enrolled after screening, and 2 participants dropped out before the evaluation.

By the time the TAUT project first stage of work started, the number of TAUT participants changed. From the updated information, out of 1033 subjects in the CCSMA dataset, 346 people are screened and mailed. Following this, 21 enrolled, 146 refused, 2 are unreachable, 58 are found to be deceased, 92 could not be located, 21 are out of the area, and 6 are deemed to be ineligible. The level of adoption is described using two classes of refuser/non-adopter and adopter, where the non-adopter class includes both refusers and ineligible respondents and adopter includes those who agreed (Table 3).

**Table 3:** Data dictionary used for profiling adopter and non-adopter

	Code	Frequency	Included/removed	Class
Not recruited	0.00	687	-	-
Enrolled in study	1.00	21	Included	Adopter
Unreachable	2.00	2	Removed	-
Refused by phone/letter	3.00	146	Included	Non-adopter
Deceased prior to study	4.00	58	Removed	-
Moved	5.00	-	-	-
Temporary moved	6.00	-	-	-
Cannot locate	7.00	92	Removed	-
Out of area	8.00	21	Removed	-
Ineligible	9.00	6	Included	Non-adopter

Individuals with codes 2, 4, 7, and 8 are not included in the study as they do not provide any information related to adoption. Based on this categorisation, there are 21 adopters and 152 non-adopters. In our work, the CCSMA dataset used contains personal information, details of cognitive assessments, and self-reported medical data that are collected through sessions with the PwDs and their caregivers, while the UPDB dataset contains medical history data obtained from healthcare insurers.

### Experimental set-up

For building the adoption prediction models, a range of classification algorithms are used. A set of models are built in the Weka Experimenter (University of Waikato, Version 3.7.12) using six classification algorithms: Neural Network (NN), C4.5 Decision Tree (DT), Support Vector Machine (SVM), Naïve Bayes (NB), Adaptive Boosting (AB), *k*-nearest neighbour (*k*NN), and Classification and Regression Trees (CART). Table 4 details the parameters used to train each model.

**Table 4:** Parameters used to train each model

Algorithm	Weka	Parameters used to train each model	
NN	Multilayer Perceptron	normalizeAttributes = True batchSize = 100 decay = False validationSetSize = 0 trainingTime = 500	resume = False autoBuild = True normalizeNumericClass = True learningRate = 0.3 reset = True
C4.5 DT	J48	seed = 1 unpruned = False confidenceFactor = 0.25 numFolds = 3 batchSize = 100 reducedErrorPruning = False useLaplace = False	doNotMakeSplitPointActualValue = False binarySplits = False doNotCheckCapabilities = False minNumObj = 2 useMDLcorrection = True collapseTree = True
SVM	SMO	numFolds = -1 randomSeed = 1 batchSize = 100 kernel = Poly Kernel	checksTurnedOff = False filterType = Normalize training data toleranceParameter = 0.01 epsilon = 1.0E-12
NB	NaïveBayes	useKernelEstimator = False batchSize = 100	displayModelInOldFormat = False useSupervisedDiscretization = False
AB	AdaBoostM1	seed = 1 weightThreshold = 100 batchSize =100	numIterations resume = 2 useResampling = False
*k*NN	IBK	batchSize = 100 KNN = 1 distanceWeighting = No distance weighting	windowSize = 0 meanSquared = False crossValidate = False
CART	SimpleCART	seed = 1 batchSize = 100 useOneSE = False usePrune = True	numFoldsPruning = 5 minNumObj = 2.0 heuristic = True sizePer = 1.0

In the TAUT study, the number of non-adopters (152) in comparison to the number of adopters (21) is high, resulting in imbalanced data. With such imbalance in the data, there is a possibility that the model may be biased towards the majority class, i.e. the non-adopter class, leading to a higher prediction error. Conversely, such bias can cause rejection of a potential adopter and classify a PwD as a non-adopter. In these scenarios, a potential adopter could be missed and deprived of using assistive technology. To handle this imbalance between the two classes, Synthetic Minority Over-Sampling Technique (SMOTE) [26] is applied. In this method, a randomisation algorithm is used to create new samples of minority class instances.

In real-world scenarios, the data to be tested may have an imbalance. Therefore, an ideal case of evaluating model performance is to build models using rebalanced data and testing it on original data. As a best practice, in the case of imbalanced datasets, it is beneficial to train the model on SMOTE data and test it on the original data [4]. It is more likely that recruitment in other datasets also can end up in having more non-adopters than adopters. Therefore, training the model using SMOTE data and testing it on the original data facilitates testing the model performance in real scenarios, where the given dataset may have imbalances. The SMOTE data models have an equal chance to classify the new instances as adopter or non-adopter based on the used features and the robustness of the classification algorithms. Therefore, in our work, which involves imbalanced datasets, we train the model on SMOTE data for different classification algorithms and test it on the original data.

When evaluating the models, we also assess how interpretable the models are by a human. Understanding the outputs of the built models by the end-user is essential. The end-users in our case are healthcare professionals who may not have technical or computational experience. Therefore, it is necessary that built models are easily understood, interpreted, and analysed. Ease of use and outcomes should be salient features of the healthcare-based models. The outcomes of DT-based models, for example, CART and C4.5 DT, are easily understood and can be analysed. Thus, DT models are good for healthcare-based applications [4]. On the other hand, *k*NN works on finding the nearest neighbour based on the similarity between the new instances and its neighbours. This is a useful feature for healthcare professionals as they can analyse the outcome of a *k*NN-based model depending on their experience in a similar scenario with a PwD. Unlike *k*NN and DT, the outcome from complex models, such as NNs and SVMs, is difficult to interpret.

### CCSMA dataset results

Our previous work, prior to the TAUT project, evaluated the adoption of a video-based reminder system for PwDs, and ad hoc data from a representative cohort is collected in a basic clinical setting [8]. A limited set of features such as age, gender, Mini-Mental State Exam (MMSE) score, living arrangement, mobile reception, and broadband are perceived to be useful features by the research team. However, from the technology perspective and to understand individuals’ compliance with the technology, it is crucial to include medical history, education, and job details in adoption prediction [4, 11, 27]. Features like mobile reception, broadband¸ and living arrangement are obvious features essential for using technology. However, these features cannot be the primary reasons for adopting technology and providing insight into a PwD’s ability for adoption. In contrast, the CCSMA dataset has a more relevant set of features that represent data from the cognitively impaired older cohort and is obtained from long-term scientific studies [4]. The features obtained from the CCSMA can be reflective of a participant’s decision-making of long-term adoption.

The first stage of work in the TAUT project analysed 31 features from the CCSMA. These features included age, gender, employment, education, MMSE score, self-reported health conditions, and genetic details [4]. The 31-features dataset is subsequently reduced to 11 features dataset using univariate (Chi-square test) and multivariate analysis (logistic regression). Table 5 gives the details of the set of possible values that these 11 features could take in our work. Features such as age, gender, education, and job provide the background of a subject. Dementia code AD pure (padom) indicates the presence of AD or other forms of dementia. Last CCSMA observation (lastV) and CCSMA observation date (lastObs) features are believed to be some sort of measure of social inclusion and the subject’s interest in this kind of study. Heart attack, stroke, hypertension, and high cholesterol are self-reported comorbidity features that are collected through sessions with the PwDs and their caregivers. These comorbidity features define the health conditions of a subject.

**Table 5:** Details of 11 features selected from the CCSMA data [4]

Features	Details			
Gender	Male = 1, female = 2			
Age (years)	-			
Education level (educ)	0 = No education 1 to 10 grade 11 = Eleventh grade/no diploma 12 = High school diploma or GED 13 = Some college	13 = Some college 14 = 2 years of college 15 = 3 years of college 16 = College degree (B.A., B.S.) 17 = Some post-graduate work		18 = M.A., M.S. 19 = Some doctoral work 20 = Doctoral degree 97 = Refused 98 = Don’t know 99 = Missing
Job category	1 = Professor, technical, manager 2 = Clerical, sales 3 = Service	4 = Agriculture 5 = Processing 6 = Machine	7 = Bench work 8 = Structural	9 = Miscellaneous 10 = Never employed
Dementia code AD pure (padom)	1 = AD-clean 2 = AD with other dementia 3 = AD-VaD	4 = VaD without AD 5 = Other dementia 9 = anycind as of x12		10 = Screened normal 11 = Evaluated normal 99= Unable to determine
lastV	1 = v1	4 = v2	7= v3	10 = v4
lastObs	1 = v1, 2 = c1, 3 = f1, 4 = v2, 5 = c2, 6 = f2, 7 = v3, 8 = c3, 9 = f3, 10 = v4, 11 = c4, 12 = f4			
Heart attack self-reported (MI)	0 = Never (birth-lastVdxK) 1 = Prevalent (before v1)	2 = Incident (v1 to RC/Dem) 3 = Post-dementia onset		4 = During PV wave(s) 9 = Missing
Stroke self-reported (CVA)	0 = Never (birth - lastVdxK) 1 = Prevalent (before v1)	2 = Incident (v1 to RC/Dem) 3 = Post-dementia onset		4 = During PV wave(s) 9 = Missing
Hypertension self-reported (HTN)	0 = Never (birth - lastVdxK) 1 = Prevalent (before v1)	2 = Incident (v1 to RC/Dem) 3 = Post-dementia onset		4 = During PV wave(s) 9 = Missing
High cholesterol self-reported (Chol)	0 = Never (birth - lastVdxK) 1 = Prevalent (before v1)	2 = Incident (v1 to RC/Dem) 3 = Post-dementia onset		4 = During PV wave(s) 9 = Missing
* CIND = Cognitive impairment not dementia		* PV wave (s) = Periodic wave of visit		* VaD = Vascular dementia

To reduce the complexity of the built models, some of the features are recategorised such as age = {above ninety, below or equal ninety}, education {no college, college/higher}, job = {known to tech, unknown to tech}, and padom = {normal, impairment/dementia}. Comorbidity features are dichotomised as a subject that never had any such health conditions, or they are diagnosed at some stage with these comorbidities. The models are built following the process described in Section 3.3. The evaluation scenario is SMOTE model: 304 instances (adopters = 152, non-adopters = 152) and test instances: 173 instances (adopters = 21, non-adopters = 152). Two sets of results are analysed, one with all the 11 features, *V*_11_ and the other with 9 features, *V*_9_, which excluded lastV and lastObs. These two features are a measure of social inclusion, and conversely, they may not always be easily available in other datasets. Therefore, it is deemed appropriate to evaluate two feature sets, one including them and other without them. From the set of models built, the *k*NN-based model achieved the best prediction accuracy of 92.48%.

The work on the CCSMA provided more useful insight into the participant ability to adopt technologies. For example, personal features such as education level and employment influenced an individual’s ability to learn new technology. However, the CCSMA dataset mainly included psychosocial data with a limited amount of self-reported medical data. The self-proclaimed medical data in the CCSMA is easier to obtain since it comes directly from the patient. The studies have found that comorbidities can be challenging in care setting. Therefore, a richer, more accurate source of medical information could be beneficial in understanding the reasons for adoption and abandonment of technology. Therefore, in the second stage of the work, the medical history data from the UPDB dataset is included in the TAUT project.

### UPDB dataset results

Even with the essential infrastructure to support the technology, such as broadband and technical background, a PwD might not be willing to adopt a technology due to the current state of their dementia or other comorbidities [4, 5, 8]. Thus, it makes sense to incorporate more reliable medical-based features in the adoption prediction models. Comorbidity in PwDs presents a particular challenge in care, and the presence of specific comorbidities may worsen the progression of dementia [27]. To some extent, comorbidity features have an impact on determining dementia and thus influence technology adoption. Consequently, it could affect the PwD decision and their ability for technology adoption. Therefore, including reliable medical-based features is essential in understanding a PwD’s decision of adoption and the extent to which adoption is carried out. The UPDB dataset, at the University of Utah, is a rich dataset containing information about genetics, demography, epidemiology, and public health [28]. The medical history data in the UPDB dataset is obtained from healthcare insurers.

#### Initial results

In the second stage of the work, we considered data about the number of times a TAUT participant is hospitalised in the category of inpatient discharge/hospitalisations (HOSP) and ambulatory surgery (AS) from the year 1996 to 2013 for the 10 most prevalent diseases [5]. The 10 most predominant diseases considered are heart disease, cancer, stroke (cerebrovascular diseases), AD, diabetes, influenza/pneumonia, nephritis, septicaemia, and accident. Considering the huge number of features in the UPDB dataset, feature reduction is carried out by merging features such as (1) total number of hospitalisation (Total HOSP), (2) total year of ambulatory surgery (Total AS), (3) total year of all 10 disease (HOSP+AS), (4) recent 3 years of HOSP, (5) recent 3 years of AS, and (6) recent 3 years for all disease (HOSP+AS). The feature reduction process resulted in a total of 24 features, giving information of the number of times a participant is hospitalised in each category. Table 6 details the 24 features from the UPDB used in our work. Using these 24 features from the UPDB dataset, a set of models are built using the process described in Section 3.3. The best prediction accuracy of 85.80% is achieved for the *k*NN-based model [5].

**Table 6:** Details of the features considered from the UPDB dataset

Feature	Feature details and labels	
Total HOSP	Number of times a subject is hospitalised in the HOPS category for any of the 10 diseases in between the year 1996 and 2013	{NoneHosp, FewTimesHosp, LotHosp}
Total AS	Number of times a subject is hospitalised in the AS category for any of the 10 diseases in between the year 1996 and 2013	{NoneAS, FewAS, LotAS}
Total Heart Total Cancer Total Chronic Total Accident Total Stroke Total AD Total Diabetes Total Influenza Total Nephritis Total Septicemia	Number of times a subject is hospitalised in the HOSP and/or AS category for each of these diseases in between the year 1996 and 2013	{NoneHeart, FewHeart, LotHeart} {NoneCancer, VisitedForCancer} {NoneChronic, VisitedChronic} {NoneAccident, VisitAccident} {NoneStroke, VisitedStroke} {NoneAD, VisitAD} {NoneDiabetes, VisitDiabetes} {NoneInfluenza, VisitInfluenza} {NoneNephritis, VisitNephritis} {NoneSepticemia, VisitSepticemia}
Recent 3 years of HOSP	Number of times a subject is hospitalised in the HOSP category in between the year 2010 and 2013	{NoneHospRecent, VisitHospRecent}
Recent 3 years of AS	Number of times a subject is hospitalised in the AS category in between the year 2010 and 2013	{NoneASRecent, VisitASRecent}
Heart_recent3Years Cancer_recent3Years Chronic_recent3Years Accident_recent3Years Stroke_recent3Years AD_recent3Years Diabetes_recent3Years Influenza_recent3Years Nephritis_recent3Years Septicemia_recent3Years	Number of times a subject is hospitalised in the HOSP and/or AS category for each of these diseases in between the year 2010 and 2013	
	{NoneHeartRecent, VisitHeartRecent} {NoneCancerRecent, VisitedForCancerRecent} {NoneChronicRecent, VisitedChronicRecent} {NoneAccidentRecent, VisitAccidentRecent} {NoneStrokeRecent, VisitedStrokeRecent} {NoneADRecent, VisitADRecent}	{NoneDiabatesRecent, VisitDiabatesRecent} {NoneInfluenzaRecent, VisitInfluenzaRecent} {NoneNephritisRecent, VisitNephritisRecent} {NoneSepticemiaRecent, VisitSepticemiaRecent}

The following sections describe how this previous work has been extended within the current study. In the current work, first, the feature selection is carried out on the 24 features UPDB dataset. Following this, the CCSMA and UPDB features are merged. Feature selection is carried out on the merged dataset to find the subset of features that are more likely to be associated with the adoption. Finally, the adoption model is built using the selected CCSMA and UPDB dataset features.

#### Feature selection

The UPDB dataset has a large number of features, and there is a possibility that not all the features may contribute to adoption prediction. It is to be noted that if these features have too many categories. The resulting model will be complex and vary according to the number of occurrences [29]. The resulting model can be viewed as complicated and impractical for real-time implementations. Additionally, it is also possible that the classification algorithms may not scale up to the large size of the feature set. Therefore, it is necessary to do a feature reduction process and remove noisy and/or irrelevant features. Feature analysis is carried out at different levels: (1) univariate analysis for selecting features that directly relate to adoption prediction and (2) multivariate analysis for finding features that indirectly relate to adoption prediction, i.e. features that may not have come up in univariate analysis but as a set of features may expressively affect adoption prediction due to interdependencies between these features.

A pair-wise Chi-squared feature selection test is performed to identify associations between the features and the output class. From this analysis, only three features, Total_AS (0.02), Total_Stroke (0.01), and Stroke_recent3Years (0.05), are found to have *p*-values less than 0.05. Following a multivariate approach, stepwise regression with backward elimination is applied. Stepwise regression is a commonly used approach for feature selection, where the features are eliminated or added from the model depending on whether it is a backward-step or forward-step approach [30]. Logistic regression is an influential statistical technique for modelling binomial outputs, i.e. the output class can take up “value of either 0 or 1 only” [31]. Before applying the stepwise regression, the 24 features UPDB dataset with 21 adopters and 152 non-adopters is rebalanced using SMOTE. The adopter minority class is boosted and made equivalent to the non-adopter majority class. A logistic regression model with backward elimination is applied to the new rebalanced 24 feature UPDB dataset. Following the feature selection, the final logistic regression model is built with 9 features detailed in Table 7.

**Table 7:** Final logistic regression parameters obtained

Variable	Model Log Likelihood	Change in -2 Log Likelihood	df	Sig. of the change
Total AS	−143.348	30.745	2	.000
Total Chronic	−129.676	3.401	1	.065
Total Stroke	−144.557	33.163	1	.000
Total AD	−130.388	4.826	1	.028
Total Diabetes	−135.937	15.924	1	.000
Total Influenza	−129.865	3.780	1	.052
Total Nephritis	−136.354	16.757	1	.000
Hosp_recent	−130.377	4.804	1	.028
AS_recent	−129.577	3.203	1	.073

Following the feature selection process, different models are built using varying feature sets. Three sets of models are built: (1) models with all the 24 features, (2) models with 3 features obtained from univariate analysis, and (3) models with 9 features obtained from multivariate analysis. Table 8 details the average prediction accuracy obtained for different models built using SMOTE data and tested on the original data for different feature sets using a range of classification algorithms.

**Table 8:** Average prediction accuracies (%) of the models obtained for 24, univariate analysis, and multivariate analysis feature sets

Dataset	NN	C4.5 DT	SVM	NB	AB	*k*NN	*CART*
All 24 features	84.02	74.56	72.78	59.17	66.86	85.80	77.25
3 features from univariate analysis	59.76	59.6	58.58	63.90	63.90	59.76	59.76
9 features from multivariate analysis	75.74	71.01	69.23	58.58	66.86	76.33	71.0

From the results presented in Table 8, it can be seen that all the models built using the complete 24 feature set have a better prediction accuracy in comparison to the models obtained using the features from the univariate and multivariate analysis. The results obtained indicated that it is better to continue with all the 24 features from the UPDB dataset.

### CCSMA and UPDB data merged

The CCSMA dataset features psychosocial information, whereas the UPDB dataset features medical-related information of the participant. Therefore, as a next logical step in the work, it would be useful to combine these features together. Besides, the accuracy obtained on the UPDB dataset is lower than that of the CCSMA dataset. A possible explanation could be that the medical history data alone cannot determine the reasons of rejection or adoption. On the other hand, the results on the CCSMA dataset indicated that incorporating personal and background information in the prediction model gave a higher prediction accuracy. Therefore, to further enhance understanding of the adoption or rejection decision, the psychosocial features from the CCSMA and medical history data from UPDB are integrated. The 11 features identified in the CCSMA dataset are merged with the 24 features from the UPDB dataset. It is hypothesised that the use of medical history data from the UPDB dataset is likely to enhance the coverage and scope of our work on adoption prediction modelling.

The CCSMA dataset is collected through the sessions with the PwDs and their caregivers, in contrast to the UPDB dataset which is obtained from healthcare insurers. The features padom, heart attack, and stroke in the CSSMA dataset have corresponding features in the UPDB. Consequently, the features padom, heart attack, and stroke from the CCSMA dataset are dropped out, and AD, heart attack, and stroke features from the UPDB dataset are considered in the merged dataset. The new dataset, CCSMA+UPDB, consists of 32 features obtained by merging 8 features from the CCSMA and 24 features from the UPDB dataset.

#### Model building

A range of analysis is carried out to find the best subset of features that could predict adoption while keeping the feature set low. As discussed, previously, lastV and lastObs features are measures of social inclusion and the subject’s interest in this kind of study. However, these two variables may not always be available; therefore, we carried out a set of analysis one including these two features and another excluding them. The evaluation scenario is SMOTE model: 304 instances (adopters = 152, non-adopters = 152) and test instances: 173 instances (adopters = 21, non-adopters = 152). Six sets of models are built: (1) all the 32 features from the CCSMA+UPDB (including lastV and lastObs features, Table 9), (2) 30 features from the CCSMA+UPDB (excluding lastV and lastObs features, Table 9), (3) 20 features, removing 10 features of recent years of admission for HOPS, AS, and different diseases (including lastV and lastObs features, Table 10), (4) 18 features, removing 10 features of recent years of admission for HOPS, AS, and different diseases (excluding lastV and lastObs features, Table 10), (5) 20 features, removing 10 features of total years of admission for HOPS, AS, and different diseases (including lastV and lastObs features, Table 11), and (6) 18 features, removing 10 features of total years of admission for HOPS, AS, and different diseases (excluding lastV and lastObs features, Table 11). The models built with 32 features and 20 features (excluding 10 features of recent years of admission for HOPS, AS, and different diseases) have nearly a similar prediction accuracy. Keeping in mind accuracy verses scalability, the 20 feature set and its corresponding 18 feature set-based model (excluding lastV and lastObs features) are considered as the best feature set for modelling adoption, while keeping the feature set low and gaining descent prediction accuracy. From the results obtained, in Table 10, it can be inferred that both the psychosocial and medical history features are crucial in determining adoption. The *k*NN-based model gave the best prediction accuracy as 99.4083%, whereas NN gave the second-best accuracy of 94.08%.

**Table 9:** Average prediction accuracies, F-Measure, and ROC area for 32 and 30 features CCSMA+UPDB dataset

32 features (lastObs and lastV included)				30 features (lastObs and lastV excluded)	
Algorithm	Results				
NN	Avg. prediction accuracy = 93.4911%			Avg. prediction accuracy = 95.2663%	
	Class	F-Measure	ROC area	F-Measure	ROC area
	Refuser	0.961	0.821	0.971	0.886
	Adopter	0.792	0.821	0.862	0.886
	Weighted avg.	0.931	0.821	0.952	0.886
C4.5 DT	Avg. prediction accuracy = 88.1657%			Avg. prediction accuracy = 88.1657%	
	Class	F-Measure	ROC area	F-Measure	ROC area
	Refuser	0.928	0.905	0.927	0.903
	Adopter	0.677	0.905	0.688	0.903
	Weighted avg.	0.883	0.905	0.884	0.903
SVM	Avg. prediction accuracy = 84.6154%			Avg. prediction accuracy = 84.0237%	
	Class	F-Measure	ROC area	F-Measure	ROC
	Refuser	0.907	0.724	0.904	0.707
	Adopter	0.552	0.724	0.526	0.707
	Weighted avg.	0.844	0.724	0.837	0.707
NB	Avg. prediction accuracy = 66.2722%			Avg. prediction accuracy = 67.4556%	
	Class	F-Measure	ROC area	F-Measure	ROC
	Refuser	0.769	0.641	0.779	0.635
	Adopter	0.374	0.641	0.382	0.635
	Weighted avg.	0.699	0.641	0.709	0.635
AB	Avg. prediction accuracy = 75.7396%			Avg. prediction accuracy = 75.7396%	
	Class	F-Measure	ROC area	F-Measure	ROC
	Refuser	0.842	0.725	0.842	0.728
	Adopter	0.481	0.725	0.481	0.728
	Weighted avg.	0.778	0.725	0.778	0.728
*k*NN	Avg. prediction accuracy = 99.4083%			Avg. prediction accuracy = 97.0414%	
	Class	F-Measure	ROC area	F-Measure	ROC
	Refuser	0.996	1.000	0.982	0.997
	Adopter	0.983	1.000	0.918	0.997
	Weighted avg.	0.994	1.000	0.971	0.997
*CART*	Avg. prediction accuracy = 85.2071%			Avg. prediction accuracy = 84.6154%	
	Class	F-Measure	ROC area	F-Measure	ROC
	Refuser	0.912	0.802	0.909	0.765
	Adopter	0.545	0.802	0.500	0.765
	Weighted avg.	0.847	0.802	0.836	0.765

**Table 10:** Average prediction accuracies, F-Measure, and ROC area for 20 and 18 features CCSMA+UPDB dataset (recent years’ disease set removed from UPDB)

20 features (lastObs and lastV included)—recent years disease set removed from UPDB				18 features (lastObs and lastV excluded)—recent years disease set removed from UPDB	
Algorithm	Results				
NN	Avg. prediction accuracy = 94.0828%			Avg. prediction accuracy = 97.0414%	
	Class	F-Measure	ROC area	F-Measure	ROC area
	Refuser	0.965	0.830	0.982	0.969
	Adopter	0.821	0.830	0.918	0.969
	Weighted avg.	0.939	0.830	0.971	0.969
C4.5 DT	Avg. prediction accuracy = 86.9822%			Avg. prediction accuracy = 86.9822%	
	Class	F-Measure	ROC area	F-Measure	ROC area
	Refuser	0.920	0.892	0.920	0.892
	Adopter	0.645	0.892	0.645	0.892
	Weighted avg.	0.871	0.892	0.871	0.892
SVM	Avg. prediction accuracy = 83.432%			Avg. prediction accuracy = 84.0237%	
	Class	F-Measure	ROC area	F-Measure	ROC
	Refuser	0.900	0.703	0.904	0.707
	Adopter	0.517	0.703	0.526	0.707
	Weighted avg.	0.832	0.703	0.837	0.707
NB	Avg. prediction accuracy = 72.1893%			Avg. prediction accuracy = 72.1893%	
	Class	F-Measure	ROC area	F-Measure	ROC
	Refuser	0.820	0.672	0.820	0.668
	Adopter	0.390	0.672	0.390	0.668
	Weighted avg.	0.744	0.672	0.744	0.668
AB	Avg. prediction accuracy = 75.7396%			Avg. prediction accuracy = 75.7396%	
	Class	F-Measure	ROC area	F-Measure	ROC
	Refuser	0.842	0.725	0.842	0.728
	Adopter	0.481	0.725	0.481	0.728
	Weighted avg.	0.778	0.725	0.778	0.728
*k*NN	Avg. prediction accuracy = 99.4083%			Avg. prediction accuracy = 97.0414%	
	Class	F-Measure	ROC area	F-Measure	ROC
	Refuser	0.996	1.000	0.982	0.997
	Adopter	0.983	1.000	0.918	0.997
	Weighted avg.	0.994	1.000	0.971	0.997
*CART*	Avg. prediction accuracy = 86.9822%			Avg. prediction accuracy = 84.0237%	
	Class	F-Measure	ROC area	F-Measure	ROC
	Refuser	0.922	0.834	0.904	0.767
	Adopter	0.607	0.834	0.526	0.767
	Weighted avg.	0.866	0.834	0.837	0.767

**Table 11:** Average prediction accuracies, F-Measure, and ROC area for 20 and 18 features CCSMA+UPDB dataset (total years’ disease set removed from UPDB)

20 features (lastObs and lastV included)—total years disease set removed from UPDB				18 features (lastObs and lastV excluded)—total years disease set removed from UPDB	
Algorithm	Results				
NN	Avg. prediction accuracy = 87.574%			Avg. prediction accuracy = 84.0237%	
	Class	F-Measure	ROC area	F-Measure	ROC area
	Refuser	0.922	0.894	0.900	0.831
	Adopter	0.696	0.894	0.597	0.831
	Weighted avg.	0.882	0.894	0.847	0.831
C4.5 DT	Avg. prediction accuracy = 76.9231%			Avg. prediction accuracy = 75.7396%	
	Class	F-Measure	ROC area	F-Measure	ROC area
	Refuser	0.853	0.711	0.845	0.701
	Adopter	0.466	0.711	0.438	0.701
	Weighted avg.	0.784	0.711	0.773	0.701
SVM	Avg. prediction accuracy = 76.9231%			Avg. prediction accuracy = 78.1065%	
	Class	F-Measure	ROC area	F-Measure	ROC
	Refuser	0.853	0.690	0.861	0.697
	Adopter	0.466	0.690	0.479	0.697
	Weighted avg.	0.784	0.690	0.794	0.697
NB	Avg. prediction accuracy = 60.355%			Avg. prediction accuracy = 61.5385%	
	Class	F-Measure	ROC area	F-Measure	ROC
	Refuser	0.722	0.597	0.730	0.598
	Adopter	0.309	0.597	0.330	0.598
	Weighted avg.	0.649	0.597	0.659	0.598
AB	Avg. prediction accuracy = 73.3728%			Avg. prediction accuracy = 73.3728%	
	Class	F-Measure	ROC area	F-Measure	ROC
	Refuser	0.833	0.688	0.833	0.688
	Adopter	0.348	0.688	0.348	0.688
	Weighted avg.	0.747	0.688	0.747	0.688
*k*NN	Avg. prediction accuracy = 89.9408%			Avg. prediction accuracy = 85.7988%	
	Class	F-Measure	ROC area	F-Measure	ROC
	Refuser	0.939	0.969	0.911	0.939
	Adopter	0.721	0.969	0.647	0.939
	Weighted avg.	0.900	0.969	0.864	0.939
*CART*	Avg. prediction accuracy = 79.8817%			Avg. prediction accuracy = 78.1065%	
	Class	F-Measure	ROC area	F-Measure	ROC
	Refuser	0.874	0.773	0.862	0.766
	Adopter	0.500	0.773	0.464	0.766
	Weighted avg.	0.808	0.773	0.792	0.766

#### Wrapper method feature selection

The final feature set obtained from the merge of psychosocial information from the CCSMA and the medical history information from the UPDB resulted in 20 features; however, the final number of features obtained is still large. Therefore, to carry out further feature reduction, a wrapper method is applied as part of the feature selection process. A wrapper method is a search problem that finds the subset of features from a given set of features for a predefined learning algorithm [32]. The method finds the best subset of features by building different models using each subset of features and comparing the performance of each of the models built.

In our case, the *k*NN classifier gave the best prediction accuracy as 99.4083%, whereas NN gave the second-best accuracy of 94.08% with the 20 feature set. Considering the complexity of NN models, *k*NN is used as a preferred learning algorithm in the Wrapper method with 20 feature SMOTE data. The Wrapper feature selection method resulted in 15 features: age, gender, education, job, lastObs, Hypertension, High Cholesterol, Total HOSP, Total AS, Total Cancer, Total Chronic, Total Accident, Total Stroke, Total Diabetes, and Total Nephritis. Using this 15 feature set, a *k*NN-based model is built using SMOTE data and tested on the original data. From this work, 98.22% accuracy is achieved.

#### Statistical significance

For finding statistically significant differences between models built using CCSMA, UPDB, and CCSMA+UPDB approaches, a one-way ANOVA test is carried out [33]. To find significant evidence of a difference between the models built, three test cases (CCSMA, UPDB), (CCSMA+UPDB, CCSMA), and (CCSMA+UPDB, UPDB) are considered, and the average prediction accuracies shown in Table 12 is used. In comparison to the results obtained for the CCSMA and the UPDB datasets, the combined feature sets have an improved prediction accuracy.

**Table 12:** Average prediction accuracies (%) for models built using CCSMA, UPDB, and CCSMA+UPDB combined

	CCSMA (11 features)	UPDB (24 features)	CCSMA+UPDB (20 features)
*NN*	90.75	84.02	94.08
*DT*	84.97	74.56	86.98
*SVM*	72.83	72.78	83.43
*NB*	46.82	59.17	72.19
*AB*	68.79	66.86	75.74
*kNN*	92.48	85.8	99.41
*CART*	87.28	77.25	86.98

First, the significant evidence of a difference between methods CCSMA and UPDB is tested. An ANOVA table (Table 13) is generated using the values of columns CCSMA and UPDB from Table 12. In Table 13, Df, SS, MS, F, and *p* columns represent sum of squares, degrees of freedom, mean squares, and test statistics and *p* value, respectively. The other two tests, (CCSMA+UPDB, CCSMA) and (CCSMA+UPDB, UPDB), are also run, and the corresponding *p* values are obtained as shown in Table 12. Based on the *p* values of 0.000918, 0.00432, and 0.000085, we can conclude that there is statistically significant evidence of a difference between CCSMA, UPDB, and CCSMA+UPDB approaches.

**Table 13:** ANOVA table for CCSMA, UPDB, and CCSMA+UPDB methods significance test

		Df	SS	MS	F	*p*
ANOVA table for CCSMA and UPDB	Accuracy_all$UPDB	1	1442.7	1442.7	48.97	0.000918
	Residuals	5	147.3	29.5		
ANOVA table for CCSMA+UPDB and CCSM	Accuracy_all$CCSMA_UPDB	1	1442.7	1442.7	48.97	0. 00432
	Residuals	5	147.3	29.5		
ANOVA table for CCSMA+UPDB and UPDB	Accuracy_all$CCSMA+UPDB	1	503.3	503.3	133.9	0.000085
	Residuals	5	18.8	3.8		

## Discussion

We aimed to minimise the likelihood of failure of assistive technologies by analysing the potential user’s background prior to recommending a technology. Keeping this view in mind, a prediction tool is to be implemented within TAUT that classifies adopters from non-adopters based on a reminder app usage and set of features including psychosocial and medical history data. The TAUT project deals with three sets of information: (1) information relating to individuals’ compliance with the usage of the TAUT reminder app, (2) details of user’s personal and background information from the CCSMA, and (3) medical history data from the UPDB. Therefore, the work is carried out in different stages.

The summary table, shown in Table 1, compared the TAUT approach to other existing models and theories related to adoption modelling. The TAM model predicts technology acceptance in general based on usefulness and ease of use features. PIADS extends TAM by adding personal and external factors. UTAUT improves both of these approaches by including more features. STAM is developed to understand mobile phone usage and adoption in older patients. In comparison to these models, the TAUT approach is more data-driven, incorporates a varied range of features, represents cognitively impaired older cohort, and experimentally evaluated. The TAUT participants represent the actual population, and the data comes from long-term scientific studies. It is envisioned to build the final adoption model using data related to participants’ compliance with reminder app usage and details of psychosocial and medical history data.

**Fig. 3:**
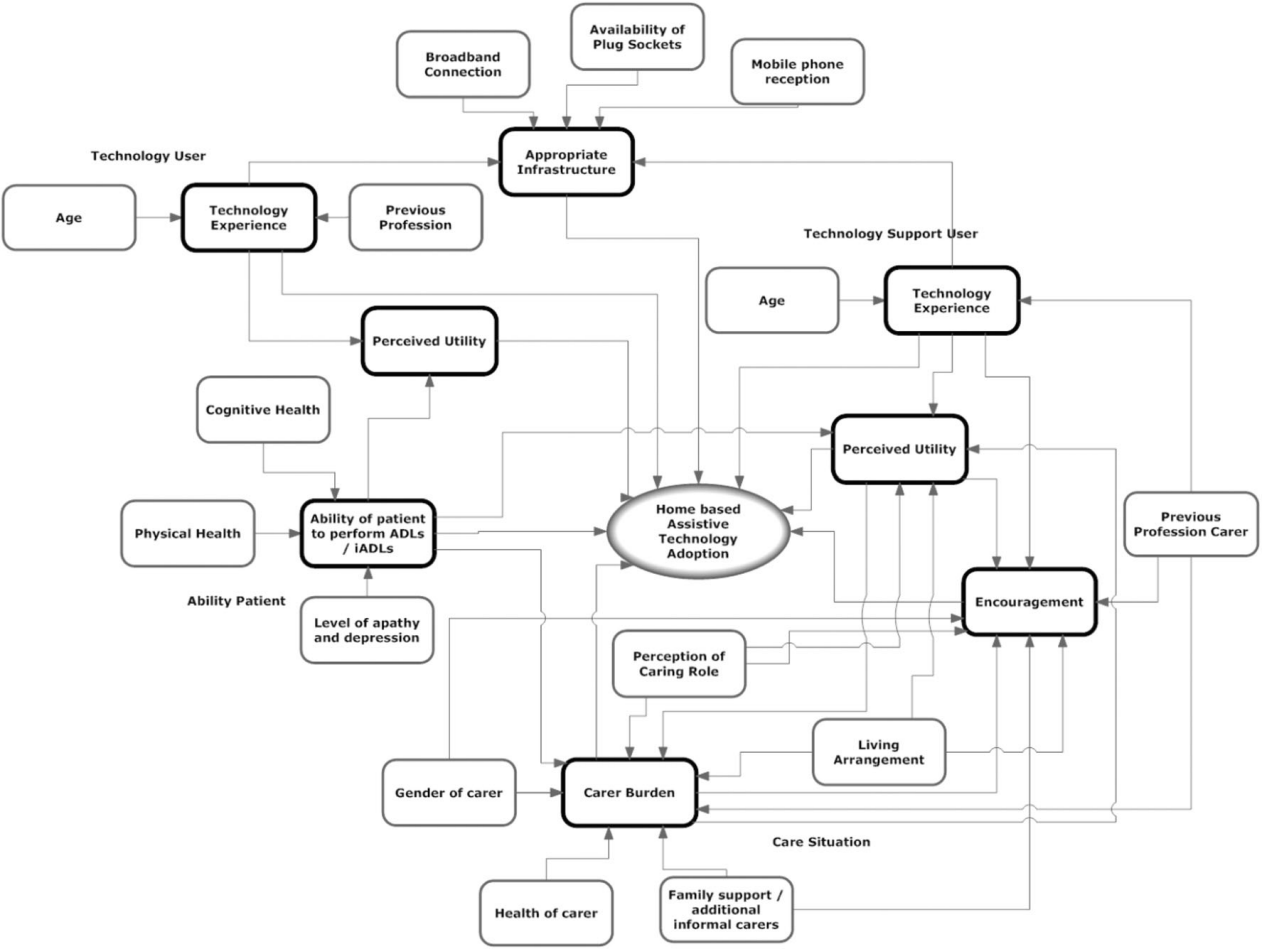
Influence diagram of features impacting on technology adoption [8]

Prior to the TAUT project, we worked on similar guidelines to that of TAM and investigated features that are mostly related to personal and environmental settings of a PwD [8]. In a basic clinical setting, ad hoc data are collected through interviews with the research team comprising of computer scientists, biomedical engineers, geriatric consultants, and research nurses. Based on the collected data, an influence diagram is created as shown in Fig. 3 [8].

As can be seen from Fig. 3, there is a relationship between the technology adoption and the identified set of features. In the influence diagram (Fig. 3), features can be categorised as independent features (within the rectangles with thin lines) and summary features (within the rectangles with thick lines). The summary feature can be a standalone feature or could be affected by independent features. For example, in Fig. 3, the technology experience (a summary feature) is affected by the independent features age and previous profession. Features like broadband, mobile reception, living arrangement, age, gender, prior technology experience, cognitive abilities, and carer setting are perceived to be key determinants in predicting adoption success [4]. On a similar background as TAM, in this work, we obtained a limited set of features that may be helpful in facilitating technology use, but they may not be the primary reason for adoption [4]. Additionally, this work lacked data from the actual older cohort and experimental evaluation [4].

Following a more data-driven approach and finding explanatory behaviour for a PwD acceptance or rejection of technology, the TAUT project recruited participants from the CCSMA dataset for 12-month assessment of the TAUT reminder app. The subjects in the CCSMA dataset are linked to the UPDB dataset, which provided medical history data of the subjects. Both the datasets have a range of features that can approximate the underlying function, which accurately model adoption prediction and provide an insight into a PwD capability to adopt a technology. In comparison to models and theories detailed in Table 1, the CCSMA and UPDB have more relevant features related to an individual’s psychosocial and medical history data, respectively. The data pertaining to these datasets come from longitudinal scientific studies and represent the actual population. In the CCSMA study, every 3 years, periodic waves of visits are performed. The medical information in the UPDB is updated annually with complete reporting of medical information.

**Fig. 4:**
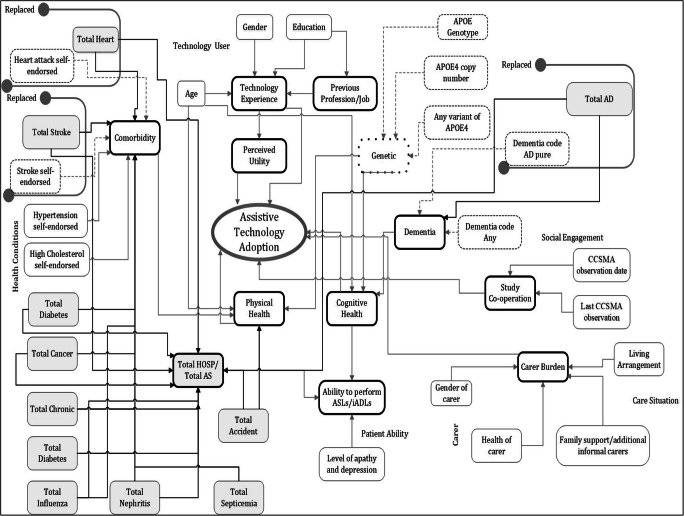
Influence diagram based on the combined features from the CCSMA and UPDB datasets

The TAUT project deals with three different types of datasets, and the final prediction model will incorporate features from app usage, CCSMA, and UPDB. The first stage of work analysed CCSMA, and from this, 11 feature-based adoption prediction model is built, achieving an accuracy of 92.48% [4]. In the second stage, an adoption prediction model is built using all the 24 features of the UPDB dataset, achieving an accuracy of 85.80% [5]. The current stage of work, reported in this paper, involves feature selection on UPDB and merge of CCSMA and UPDB features. Figure 4 shows the new influence diagram obtained by merging CCSMA and UPDB features. The CCSMA features are shown in white-rounded rectangles, and the UPDB features are in grey-shaded-rounded rectangles. In Fig. 4, the CCSMA features replaced by the UPDB features are shown within the container boxes. Figure 4 also shows a summary feature, carer burden, which may have an impact on technology adoption. Compared with the influence diagram in Fig. 3, the feature, previous profession/job, is changed from an independent feature to a summary feature in the new influence diagram. The education feature is likely to impact on the previous profession; however, the previous profession is a feature in itself.

The features last CCSMA observed and CCSMA observed date provide details about an individual’s availability when the observation is taken; therefore, they are included in the new influence diagram to indicate an individual’s engagement with the study. It is likely that an individual who is well acquainted with the study, and the associated staffs, would be more likely to participate and adopt the technology. As indicated in [34], it is very important that the interviewers can convince subjects to cooperate with the survey. Based on these considerations, we include a summary feature named study co-operation, which outlines individual willingness to partake in such studies, and we believe such a parameter may be significant in an individual decision for adoption [35].

The new influence diagram shown in Fig. 4 has no information about the caregiver technology experience. This is because the CCSMA dataset has no information about the caregiver technology experience. The new influence diagram does not have any technology connectivity features (broadband and mobile reception), as they are no longer considered significant, and it would be useful to look at the features related to the subject.

In comparison to features used in theories and model detailed in summary Table 1 and Fig. 3, the features in the new influence diagram are significantly more relevant and detailed. Features like broadband and mobile reception can aid technology usage; however, they cannot be regarded as key determinants in adoption or abandonment of technology. On the other hand, padom (Dementia code AD pure) feature in the CCSMA directly indicates the existence of dementia and is suggestive of a user’s current cognitive abilities. Additionally, CCSMA features, like APOE genotype, APOE ε4 copy number, and any variant of APOE ε4, are genetic markers and indicate an influence on dementia. The subjects having these genetic markers may have lower cognitive functionality leading to inappropriateness of technologies [36, 37]. Features such as education and job might affect a user’s capability to learn and adapt to new technology. For example, if a person has worked in a clerical or technical job, they are more likely to have used technology in comparison to someone in heavy industry or retail. In the CCSMA feature analysis, it is found that age, gender, education, job, comorbidities, and dementia-related features are significant in determining the adoption or abandonment of technology [4]. The self-reported medical information from the CCSMA is replaced by more verified medical information of the UPDB, in the CCSMA, and UPDD merged dataset. The prediction accuracy obtained in the CCSMA analysis indicated that the CCSMA features are better for understanding relationships between the adoption and the abandonment of technology than the UPDB features. However, the medical data gave a useful insight into user health conditions, which is important to understand adoption failure. It is likely that users with ailing medical conditions are potential non-adopters, as reported in [4, 8, 27].

The motivation of combining psychosocial and medical history data is to approximate the underlying function that can accurately model adoption prediction. Considering scalability versus accuracy, it is necessary to consider only those features that significantly affect adoption and ignore features that have little to no effect on adoption prediction, so that the resulting model is small and accurate. With the merged set of features from the CCSMA and UPDB, 20 features *k*NN and NN based adoption prediction model gave 99.41% and 94.08% accuracy, respectively. Both these results have improved accuracy in comparison to the best accuracy achieved (92.48%) in our previous work, based on psychosocial and self-reported health data for the same cohort.

Considering the ease of use features, feature reduction is carried out to narrow down a large range of features into a smaller number that is possibly easy to collect. Further, the categorisation of the features into fewer labels makes it easy to collect, interpret, and understand, thus making the model more parsimonious. In the selected feature set, personal features such as gender, age, education, and job can be easily collected. The comorbidity features identified are labelled as either the subject has these comorbidities or does not have. Besides, the comorbidity features included in the work does not include details of what level of a medical condition a subject has. This makes it easy to collect features and input to the model for prediction. In contrast to TAM, which explains user intention to use technology, our approach is more experimentally evaluated. The TAM tool is still general and not designed for a particular profession [38]. The work described in this paper gives explanatory behaviour of participant accepting or rejecting a technology. The identified features provide an insight into a participant capability to adopt a particular technology. Table 14 detail the reasons of refusal by the participants. As can be seen from Table 13, there could be different reasons for the participants’ abandonment of technology. The reasons include subject’s inability to learn a new technology (user’s background from CCSMA such as technology experience), ailing medical conditions (current medical status from UPDB), and complexity of the app. It is to be noted that in the 20 feature set from the CCSMA+UPDB dataset, recent 3 years of HOSP, recent 3 years of AS, and recent 3 years for all disease (HOSP+AS) have been dropped. However, the recent medical history information can give useful insight for disinterest among the refusers, who initially agreed to be part of the TAUT study but later withdrew (Table 14).

**Table 14:** Reasons for refusal by the subjects in the TAUT study

Reason of refusal	
Prefers not to try to learn device and app	
Dissatisfied with reminder device; subject unable to learn new reminding tool	
Subject’s failing health; subject put on hospice	
Participant unable to learn/use technology	
The informant reported: participant cannot learn to use device because he/she cannot remember what it is when it alarms—gets nervous that it is fire alarm going off or thinks it is a remote control	
Too busy and prefers to use physical the calendar on the fridge. Would like to simplify his life	
The participant could not learn to use technology	
The participant has a hard time learning smartphone/App and does not care to use it. Prefers regular calendar. Gave satisfaction survey	
Reported “not good” at technology and did not use the device	
Due to failing health. Participant is on O2 tank 24/7 for pneumonia. Follow-up OK	
Inconvenient to carry around extra phone	
Too busy and not interested in learning new technology (smartphone or App) but might look into “simple” tablet through AARP. Okay to contact for follow-up	
Participant not interested in using or learning how to use the smartphone	
Daughter called and insisted that parents are removed from the study	

**Fig. 5:**
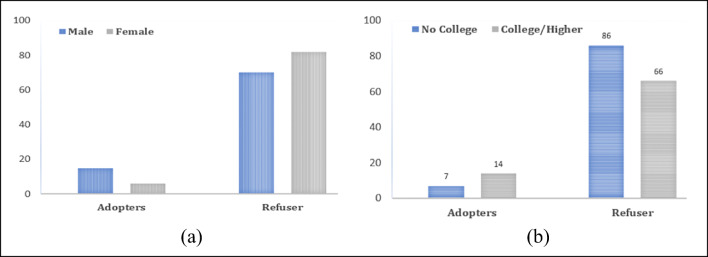
(**a**) Distribution of male and female in the adopter and refuser class. (**b**) Educational information of adopters and refuser class

Features such as education and job might affect a user’s capability to learn and adapt new technology. For example, if a person has worked in a clerical or technical job, they are more likely to have used technology in comparison to someone in heavy industry or retail. In the collected data, it is observed that gender also has significance in technology adoption. The distribution of male and female in the adopter and refuser class is shown in Fig. 5a. Figure 5b shows the distribution of educational information of adopters and refuser class. From Fig. 5b, it can be inferred that the subjects who have higher educational background are more likely to adapt to technology.

The age distribution between adopters and refusers class is shown in Table 15. For comparison of the age distribution between the two classes, the *t*-test is performed, and the *t*-value obtained is 0.91.

**Table 15:** Mean and standard deviation values for adopter and refuser class

Class	Mean	STDEV
Adopter	89.76	3.11
Refuser	90.59	3.99

## Conclusion and future work

Assistive technologies are beneficial only if they are used. Understanding the reasons that may influence adoption is crucial to the success of these technologies. The work carried out in the TAUT project gives explanatory behaviour of a PwD for adopting or rejecting a technology given the user’s background and medical history. The identified features provide insight into a PwD capability to adopt a particular technology. In summary, by combining the CCSMA and the UPDB datasets, the model can obtain a prediction that is more accurate and efficient. A substantially improved prediction accuracy is achieved using 20 features. Thus, we can conclude that a good prediction can be obtained with psychosocial data, including self-reported medical data, collected in the community (CCSMA). We can get a reasonable, but not as good, prediction from medical data from validated sources (in our case from the insurers via UPDB). However, we get excellent predictions from combining these two data sources and using medical data, from authenticated medical sources, combined with psychosocial data. The obtained feature set from this analysis is significant, as considerably high prediction accuracy is obtained and the built model can distinguish adopters from non-adopters with higher confidence. We are successful in modelling adoption from low-cost, convenient, and non-invasive features, which can prove to be beneficial in the long-term success of assistive technologies. From the work presented, it can be concluded that in general for such interventions, combining psychosocial data with health service data can improve prediction of a successful adoption.

Distinguishing adopters from non-adopters is important. A potential adopter, who can benefit from technology, should not be missed. The final stage of this work is to develop a prediction model with convenient and low-cost features that can easily screen patients. In the future, a screening tool can be developed from this work to allow prescribers of assistive technologies and family members to easily identify if a user is likely to adopt a technology or not. Given the positive results, we believe that it would be possible to predict adoption in other contexts where a similar range of features can be obtained. Future work is being undertaken to identify further differences between adopters and non-adopters by correlating features with usage patterns of the TAUT app (e.g. number set, number missed, and measures of perceived utility and usability). Another interesting work could be to investigate the adoption of different technology and use of technology in different demographic groups.
